# Air quality prediction models based on meteorological factors and real-time data of industrial waste gas

**DOI:** 10.1038/s41598-022-13579-2

**Published:** 2022-06-03

**Authors:** Ying Liu, Peiyu Wang, Yong Li, Lixia Wen, Xiaochao Deng

**Affiliations:** 1 Departments of Geosciences and Environmental Engineering, Southwest Jiaotong University, Chengdu, 610000 China; 2 IT Electronics Eleventh Design & Research Institute Scientific and Technological Engineering Corporation Limited, Chengdu, 610000 China

**Keywords:** Environmental sciences, Environmental impact, Computational science

## Abstract

With the rapid economic growth, air quality continues to decline. High-intensity pollution emissions and unfavorable weather conditions are the key factors for the formation and development of air heavy pollution processes. Given that research into air quality prediction generally ignore pollutant emission information, in this paper, the random forest supervised learning algorithm is used to construct an air quality prediction model for Zhangdian District with industrial waste gas daily emissions and meteorological factors as variables. The training data include the air quality index (AQI) values, meteorological factors and industrial waste gas daily emission of Zhangdian District from 1st January 2017 to 30th November 2019. The data from 1st to 31th December 2019 is used as the test set to assess the model. The performance of the model is analysed and compared with the backpropagation (BP) neural network, decision tree, and least squares support vector machine (LSSVM) function, which has better overall prediction performance with an RMSE of 22.91 and an MAE of 15.80. Based on meteorological forecasts and expected air quality, a daily emission limit for industrial waste gas can be obtained using model inversion. From 1st to 31th December 2019, if the industrial waste gas daily emission in this area were decreased from 6048.5 million cubic meters of waste gas to 5687.5 million cubic meters, and the daily air quality would be maintained at a good level. This paper deeply explores the dynamic relationship between waste gas daily emissions of industrial enterprises, meteorological factors, and air quality. The meteorological conditions are fully utilized to dynamically adjust the exhaust gas emissions of key polluting enterprises. It not only ensures that the regional air quality is in good condition, but also promotes the in-depth optimization of the procedures of regional industrial enterprises, and reduces the conflict between environmental protection and economic development.

## Introduction

Air quality is a critical issue related to people’s health and livelihoods, and one of the obstacles to regional economic development and social progress. In addition to air quality monitoring and management, air quality forecasting during periods of polluted weather has also become a focus of environmental management. Especially during major events and heavy pollution emergencies, timely and accurate air quality prediction and pollution source analysis can provide a decision-making basis for management departments. If the exhaust gas emissions of enterprises can be determined according to the requirements of regional ambient air indicators and meteorological conditions, and then it could guide enterprises to adjust production processes accordingly. Air pollution caused by unfavorable meteorological factors can be effectively avoided, and enterprises can expand the production of heavy pollution processes when the weather conditions are favorable. Based on air quality prediction and pollution source analysis, it is of great practical significance to make full use of meteorological conditions to coordinate the relationship between air quality and regional development.

Some scholars at home and abroad have conducted qualitative analysis research on factors affecting air quality from the perspective of the environment, society, and economic activity, considering various factors such as waste incineration, vehicle exhaust emissions, population growth, coal combustion, industrial waste gas discharge and industrial flue gas dust. These studies confirmed that air pollution results from environmental degradation that has been majorly generated from urban population growth, industrial activities, and road fleet^1^. Industrial waste gas discharge is the main cause of air pollution in developing countries^2^. Thermal power plants and manufacturing industries are the largest sources of urban air pollution^3,4^. Other studies have selected meteorological factors such as average temperature, relative humidity, visibility, wind force scale, sun exposure, and wind direction to research the correlation between these meteorological factors and air quality. Research results indicated that average temperature, relative humidity, visibility, and wind force scale are the principal factors that affect air quality^5^. The variation in pollutant emissions affects an area within a hundred-kilometers radius from the source, depending also on local meteorological and geomorphological conditions^6^.

There is also quantitative analysis of air quality based on pollutant transport, and diffusion processes. Currently, there are two main types of air quality prediction model: mechanism models and non-mechanism models. Mechanism models involve complex physical and chemical processes, which all possess great uncertainty. They require the establishment of a relatively complete emission source inventory, accurate meteorology fields, and related models of physical and chemical processes, such as pollutant transport and diffusion. Non-mechanism models, represented by statistical models and machine learning models, do not require complex pollutant boundary fields or meteorological boundary fields, nor do they need the investigation of complex mechanism processes generated by the results. This approach can determine the trend of pollution at a certain stage only by the extraction of data characteristics. Compared with mechanism models, non-mechanism models are more convenient and practical. The most commonly applied classical statistical methods mainly include linear and nonlinear models^7^, multiple regression equation^8^, time series^9^, etc. Some conventional machine learning methods that are widely used include support vector machines^10^, decision trees^11^, Bayesian networks^12^, artificial neural networks^13^, backpropagation (BP) neural networks^14^, etc. With the continuous development of artificial intelligence, deep machine learning models has been successfully implemented to forecast air quality using time series air pollutant and meteorological datasets with excellent performances^15^.

**Figure 1:**
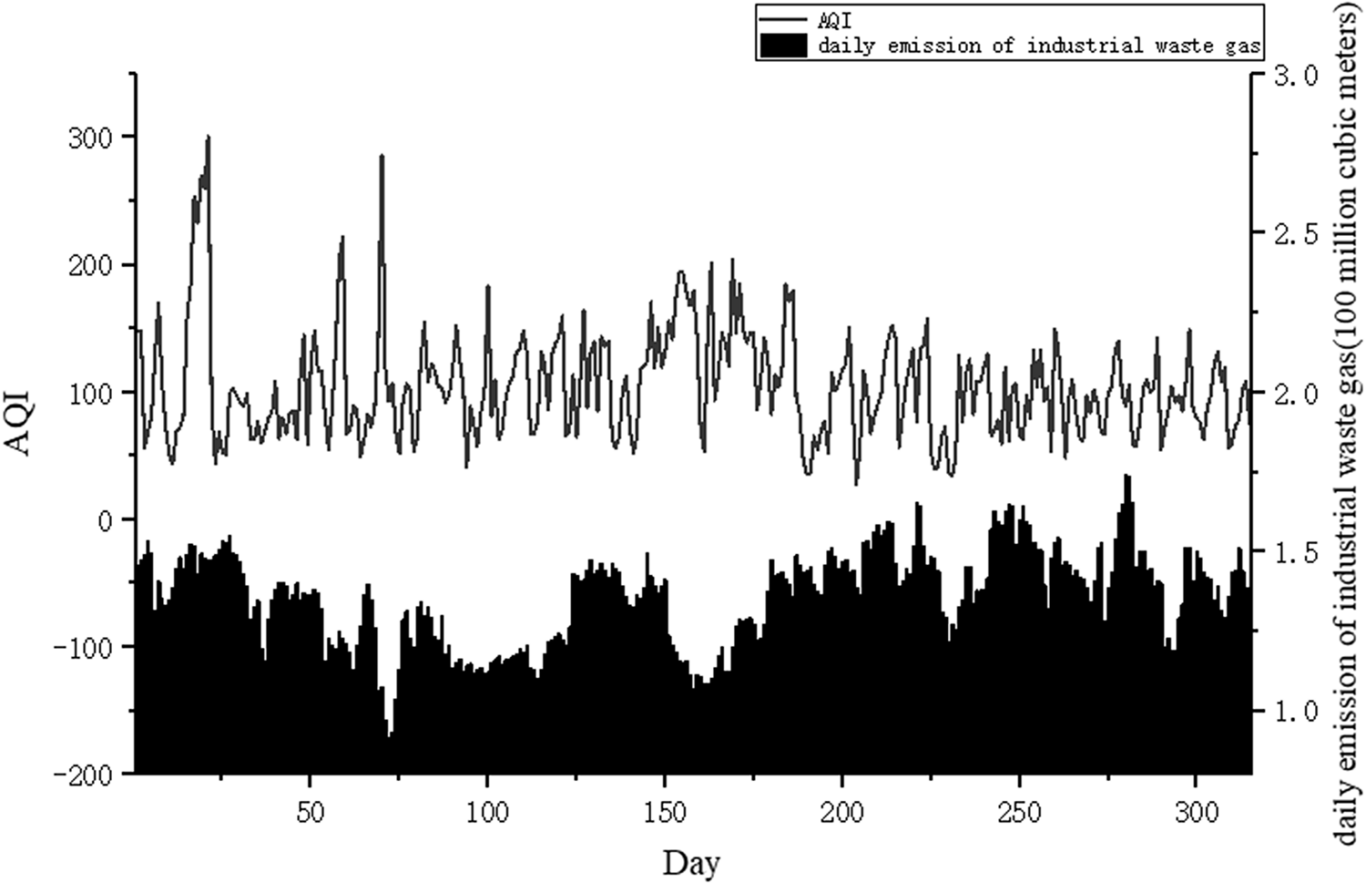
AQI and industrial waste gas emission statistics in Zhangdian District.

Some scholars also look forward to the research on air quality prediction, pointing out that the existing research on the impact of industrial waste gas emissions on air quality is qualitative analysis, and the air quality prediction research ignores the emission information of pollution sources^1–3^. Some extensive studies can be further conducted to gasses emission estimating and its impact on the surrounding environments^16^. Urban air pollution mainly comes from industry, transportation and daily life. Industrial waste gas discharge are the largest sources of urban air pollution^17^. Traffic and household emissions are relatively stable and can be regarded as constant, with little impact on fluctuations in air quality. From the daily emissions data of industrial waste gas in Zhangdian District in 2018, it can be seen that the daily emissions of industrial waste gas fluctuate greatly (Fig. 1). Air quality prediction results will inevitably be inaccurate if pollutant variable emissions are not taken into account. Individual studies use source emission inventories, which treated industrial pollutant emissions as constant^18,19^. The emission inventory of pollution sources is compiled based on the base year. According to the technical guidelines for the compilation of air pollutant emission inventory of various industries, it is mainly calculated by the emission coefficient method. The estimation is difficult to be accurate, and generally lags by 2–3 years. The data is constant and cannot be updated dynamically, and cannot reflect the impact of real-time changes in emissions from pollution sources.

Because the existing air quality prediction research ignores the real-time emission effect of industrial pollutants in the model establishment, and cannot establish a quantitative correlation between air quality and industrial pollution sources, it cannot expand the application value of air quality prediction. This study uses machine learning algorithms to an air quality forecast model by considering real-time industrial waste gas emissions and meteorological factors as variables. The current weather forecast time frame (15 days) is considered a period. During this period, according to the weather forecast, the daily emission limit of industrial pollution is determined by model inversion. By increasing or reducing the output of polluting processes or sections within the enterprise and balancing the intensity of pollution emissions, it not only ensures that the regional air environment quality remains good, but also meets the company’s supporting production requirements. It not only ensures that the environmental quality meets the standard, but also meets the normal operation of the enterprise. Regarding the selection of model algorithms, the random forest algorithm has several advantages compared with other machine learning algorithms. Firstly, the random forest algorithm can evaluate the importance of input variables and accurately predict output variables^20^ Besides, it has good anti-noise ability and does not easily fall into the problem of overfitting^21^. Finally, the random forest algorithm is suitable for modelling high-dimensional data and has strong adaptability to data sets^22^. However, a key problem with the random forest algorithm is that parameters cannot be accurately optimised. In this paper, we use the “RandomizedSearchCV” and “GridSearchCV” functions to solve this issue and realise the precise optimisation of parameters. Next, the BP neural network, decision tree, and least squares support vector machine (LSSVM) are used to compare their model performance with the random forest algorithm. To eliminate long-term cumulative systematic errors caused by factors such as inter-annual fluctuations in the number of motor vehicles, a multi-step sliding window method (using the first 365 days of data to predict the next day’s AQI) was adopted for the training set. By continuously incorporating measured data of air quality, meteorological conditions and industrial exhaust emissions into the training set and updating the training set in real time, the impact of long-term changes in traffic emissions on air quality can be reflected.

## Study area and data

**Figure 2:**
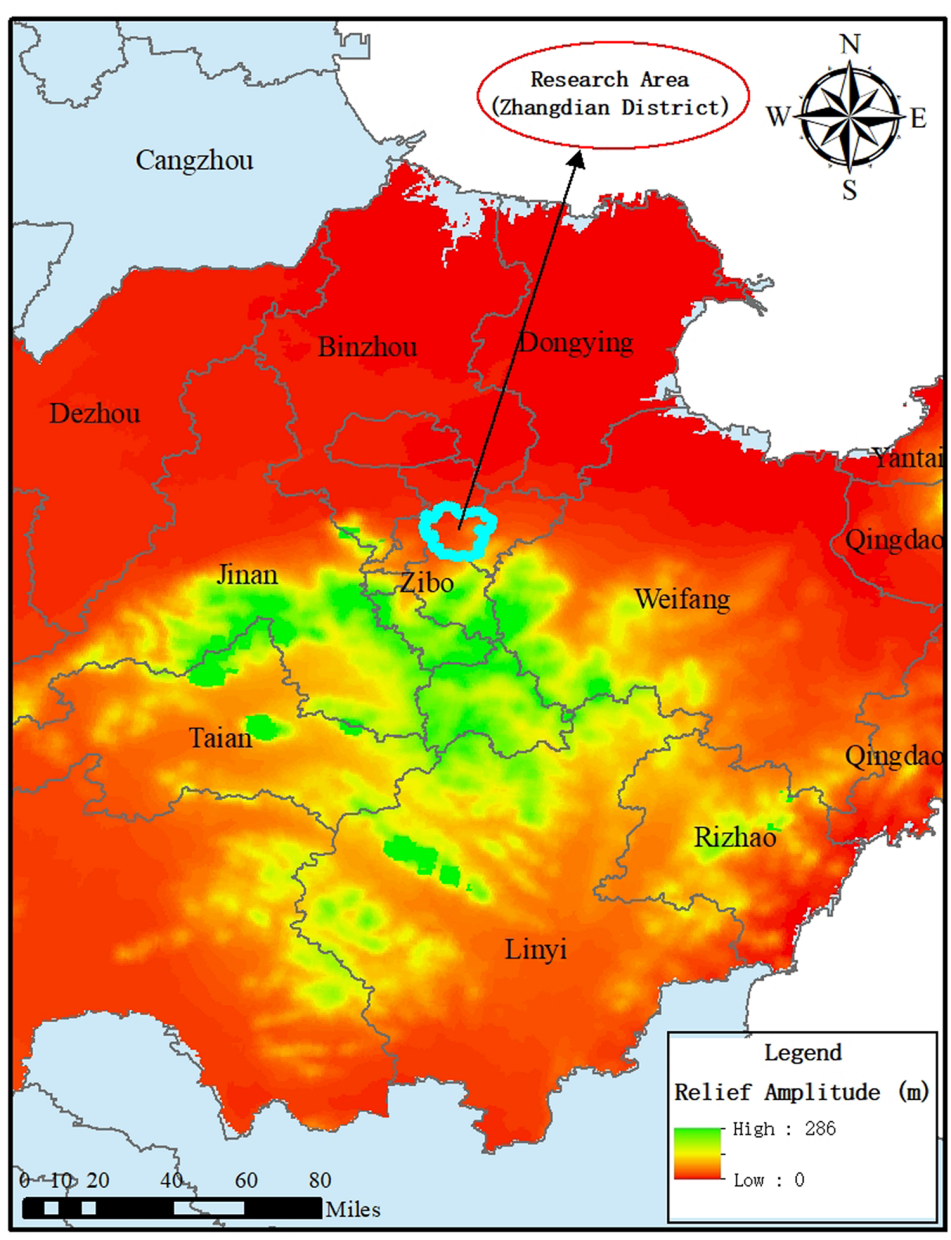
Relief amplitude of research area. The map was generated with ArcGIS10.2 (https://www.esri.com/en-us/arcgis/products/develop-with-arcgis/overview).

Zhangdian District is located in the middle of Zibo City, Shandong Province. It is located in the junction of the Shandong Zhongshan Mountains and the North Shandong Plain. It belongs to the warm temperate monsoon type semi-dry and semi-humid continental climate. Zibo City is one of the five traditional architectural ceramics production areas in China, and its architectural ceramics enterprises are mainly located in Zhangdian District. Zhangdian District has a total of 60 key industrial enterprises above designated size, including 41 non-metallic mineral products (37 building ceramics enterprises and 4 cement production enterprises), 12 chemical products manufacturing enterprises, 3 non-ferrous metal smelting and processing enterprises, and 4 other enterprises. For reference, a relief of the research area is shown in Fig. 2.

The input variables are meteorological factors and daily emissions of industrial waste gases, while the output variable is the AQI (Air Quality Index). According to the “Ambient Air Quality Index (AQI) Technical Regulations (Trial)” (HJ 633-2012), the air quality index is divided into 0–50, 51–100, 101–150, 151–200, 201–300 and greater than 300 the six levels, corresponding to the six levels of air quality (excellent, good, light pollution, moderate pollution, heavy pollution and serious pollution). Data sources are shown in Table 1. Measured data of the AQI and the daily emissions of industrial waste gases of major polluters were obtained from Zhangdian District Bureau of Ecology and Environment. Meteorological factors (precipitation, air temperature, relative humidity, wind scale, air pressure, total sunshine intensity and precipitation) were taken from the WheatA-Big Data on Agricultural Meteorology.

**Table 1:** Data sources.

Data	Data source
AQI	Zhangdian District Bureau of Ecology and Environment
Meteorological factors	WheatA—Big Data on Agricultural Meteorology
Daily emissions of industrial waste gas	Environmental Statistics Yearbook of Zhangdian District

## Methods

### Establishment of the random forest model

The random forest algorithm is a classification and regression algorithm that integrates multiple decision trees through ensemble learning^23^. First, the random forest algorithm uses the decision tree as the basic random forest classifier. Then, the second random forest classifier bagging method is used to generate the training data set and a random subspace is used to establish the classification of each strategic decision tree. The third random forest classifier randomly selects some attributes, then divides and combines the optimal attributes of each tree. The introduction of double randomisation makes it difficult for the random forest to fall into overfitting. Besides, there is diversity among classifiers, so the random forest has superior classification and regression performance^24^.

The AQI prediction model is obtained by fitting training samples. The random forest modelling process is as follows:

1. Define the AQI prediction training set, $X_{i} \to Y_{i} ,\,\left( {i = 298} \right)$. Here, *Y*_*i*_ is the real value in the random forest prediction model, which is mapped to the measured AQI value of the *i*th sample in the data. Besides, *X*_*i*_ represents meteorological factors and industrial waste gas emissions of the *i*th sample in the data. The established feature vector, $\left\{ {I_{i1} ,\,I_{i2} , \ldots I_{in} } \right\} \to X_{i}$ represents the *i*th sample to the nth impact factor.
2. Based on the training set, establish a single regression decision tree. Through the eigenvector X and its corresponding real value Y in the training sample, search for the splitting variables and splitting values. The regression decision tree divides the whole vector space into M partitions $\left\{ {R_{1} ,\,R_{2} , \ldots R_{m} } \right\}$. Any partition can be mapped to model *C*_*m*_, and the vector can be divided into two parts by the value of a feature. The expression is:
  1 $${R}_{1}\left(j,s\right)=\left\{\left(I|{I}_{j}\le s\right)\right\},$$
  2 $${R}_{2}\left(j,s\right)=\left\{\left(I|{I}_{j}>s\right)\right\}.$$
  In the above equations, j represents an impact factor and s signifies the value when splitting. The objective function of the vector space split variable and split value search is:
  3 $$z:\underset{j,s}{\mathrm{min}}\left[\underset{{c}_{1}}{\mathrm{min}}\sum_{{x}_{i}\in {R}_{1}\left(j,s\right)}{\left({y}_{i}-{c}_{1}\right)}^{2}+\underset{{c}_{2}}{\mathrm{min}}\sum_{{x}_{i}\in {R}_{2}\left(j,s\right)}{\left({y}_{i}-{c}_{2}\right)}^{2}\right].$$
  Here, z is the minimum variance of the measured AQI value, *y*_*i*_ represents the measured value of AQI in the ith sample, *x*_*i*_ is the eigenvector of the ith sample, while *c*_1_ and *c*_2_ denote the mean value of the measured AQI values in the first and second parts.
3. Construct a complete random forest model on the basis of a single decision tree, where the generated model is a multiple nonlinear regression analysis model. The predicted value of the AQI is the average value of all the predicted values of the decision trees.

**Figure 3:**
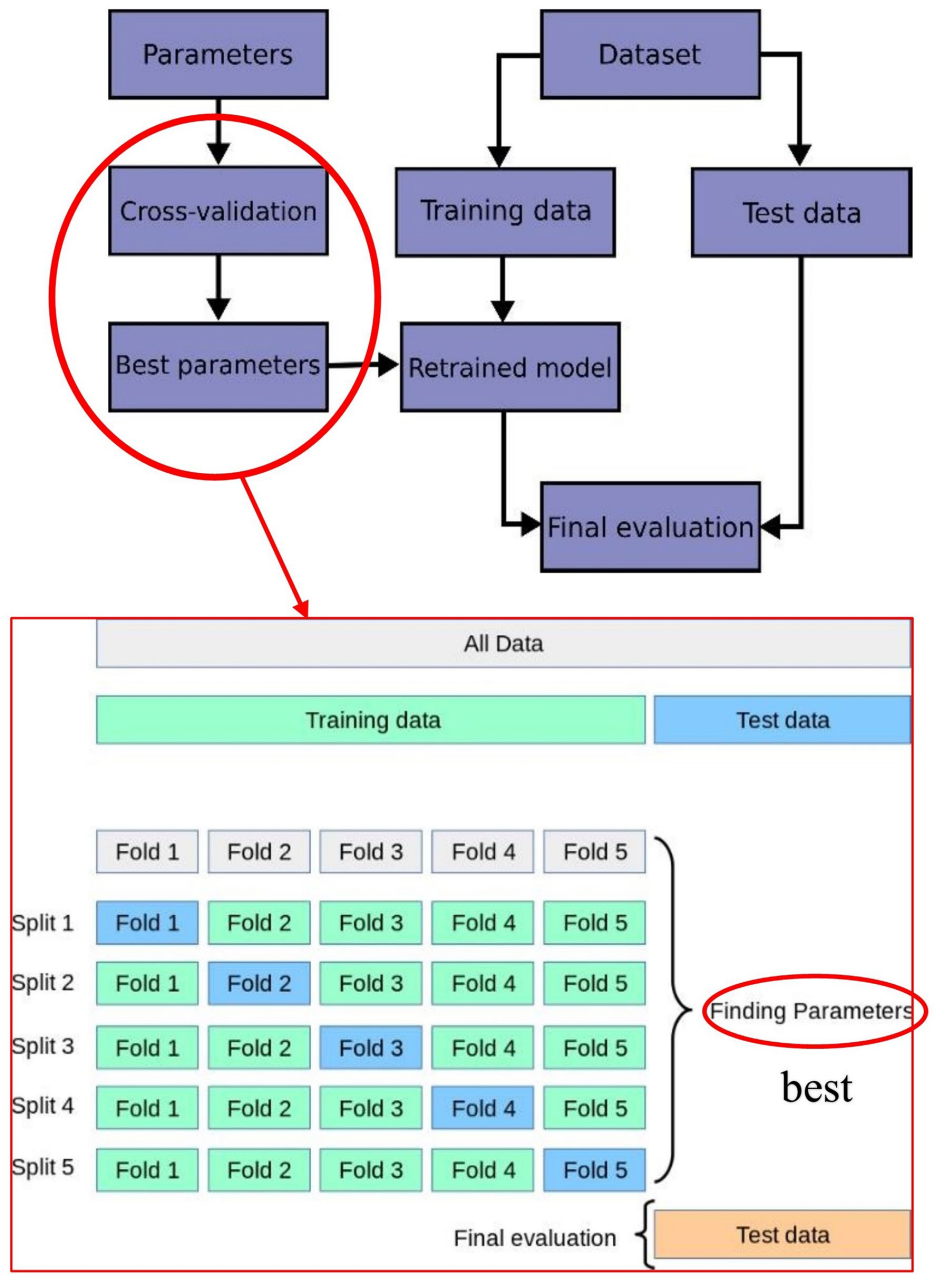
Schematic diagram of finding the optimal parameters of random forest model.

**Figure 4:**
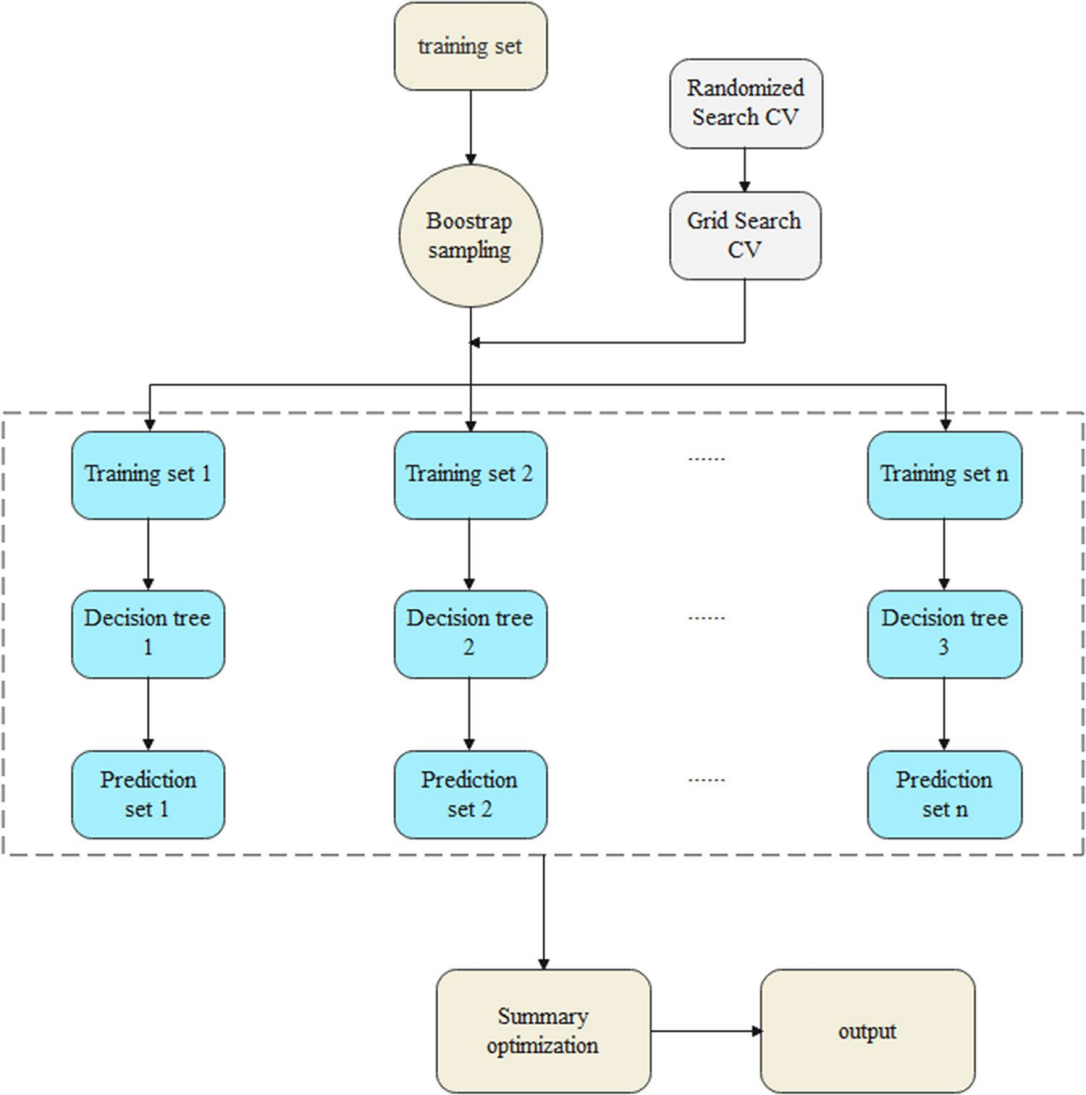
Flow chart of random forest model.

Since the random forest algorithm cannot accurately find its optimal parameters, in this paper, the model is enhanced through the “RandomizedSearchCV” and “GridSearchCV” functions to find its optimal parameters. Among them, RandomizedSearchCV is used to obtain the best parameters by randomly selecting parameter values and performing assigned times parameter combinations within the assigned parameter range; GridSearchCV is used to obtain the best parameters by exhaustively running through the given parameter values; CV is used for cross -validation, as well as parameter adjustment. Typically, RandomizedSearchCV is used first to obtain the optimal solution with a high probability of parameters, and then GridSearchCV is used to fine-tune the parameters within a certain floating range to obtain the optimal combination of parameters. “Finding Parameters” in the above figure is what RandomizedSearchCV and GridSearchCV need to do, which is to find the optimal combination of parameters. The specific process is described in Figs. 3 and 4, as follows.

### Importance evaluation of variables

The importance evaluation of variables is a vital part of the random forest algorithm. It can evaluate the influence of input variables on output variables by using the mean square residual reduction in the decision-making process of the random forest. It is the result of continuous analysis and optimisation in the training process of the random forest. Based on various permutations, the mean-square residual reduction (%IncMSE) can be used to measure the influence of corresponding independent variables and is the standard for variable importance scoring^25^. The following is the calculation method of the mean square residual:

1. Establish a regression tree for each training data set and then use this model to predict the OOB (out of bag) error. The mean square residual of b OOBs can be obtained: $MSE_{1} ,\,MSE_{2} , \ldots MSE_{b}.$
2. The number of variables selected by the self-help method in the random forest is random. Each variable *X*_*i*_ can be randomly transposed across b OOB datasets. This creates a new set of OOB tests. When the random forest regression model is used to predict the new test set, the mean square residual of the OOB after random replacement can be obtained. The matrix is as follows:
  4 $$\begin{array}{ccc}{MSE}_{11}& \cdots & {MSE}_{1b}\\ \cdots & \cdots & \cdots \\ {MSE}_{k1}& \cdots & {MSE}_{kb}\end{array}.$$
3. Next, subtract from line of the equation. Then divide the mean by the standard error to obtain the mean square residual of variable, i.e., the variable importance score. The equation is expressed as follows:
  5 $${VIM}_{i}\left(MSE\right)=\left(\frac{1}{b}\sum_{j=1}^{b}\left({MSE}_{j}-{MSE}_{ij}\right)\right)/{S}_{E},\left(1\le i\le k\right).$$

### Evaluation of model prediction accuracy

In this study, the root mean square error (RMSE), mean absolute error (MAE), and coefficient of determination (R^2^) were used for comparison between the measured and modelled AQI values^26,27^. These values can be determined as follows:

6 $$RMSE=\sqrt{\frac{\sum_{i=1}^{k}{\left(\widehat{{y}_{i}}-{y}_{i}\right)}^{2}}{n},}$$

7 $$MAE=\frac{1}{k}\sum_{i=1}^{k}\left|\widehat{{y}_{i}}-{y}_{i}\right|,$$

8 $${R}^{2}=\frac{\sum_{i=1}^{k}{\left(\widehat{{y}_{i}}-\overline{y }\right)}^{2}}{\sum_{i=1}^{k}{\left({y}_{i}-\overline{y }\right)}^{2}}.$$

In the above equations, $\widehat{y}_{i}$ represents the AQI forecast of the *i*th sample, $y_{i}$ is the measured AQI value of the *i*th sample, $\overline{y}$ denotes the average measured AQI value in all samples, and *k* is the sample size of the corresponding sample (k = 298).

## Results

### AQI and variation trend analysis

**Figure 5:**
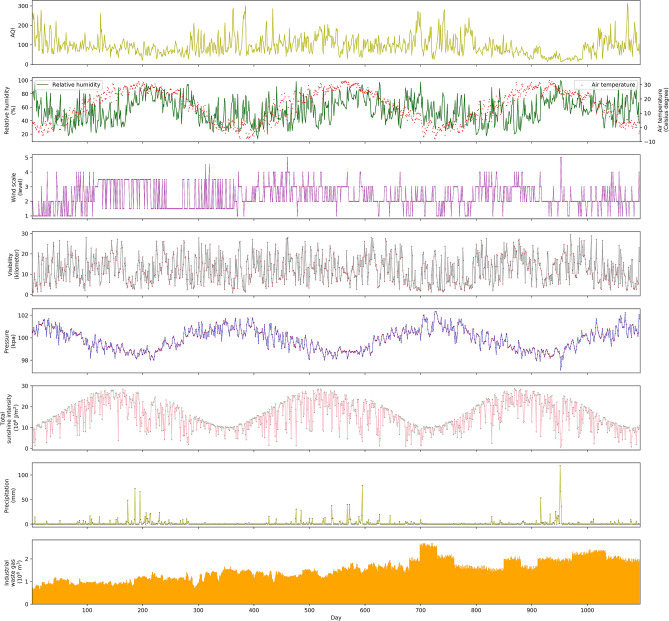
Trends of meteorological factors, industrial waste gas emissions, and AQI.

The graphs in Fig. 5 illustrate how meteorological factors, daily industrial waste gas emissions, and AQI varied in Zhangdian District from 1st January 2017 to 31th December 2019. It can be seen that the period with the largest variations in AQI was from December to March, as there are multiple peaks during this time. The minimum value of AQI during these three months was 13 while the maximum AQI value was 313. Between June and August, there were also significant variations in the AQI. In the other months, the range of change was relatively low, with the AQI remaining around 90. The relative humidity fluctuated greatly from February to May, with an average of 46.2%. However, between July and August, the relative humidity only varied slightly, with an average value of 73.7%. The average temperature in February was the lowest, then from March to August it rose slowly, while from August to October it gradually decreased. The wind scale was relatively stable, although in March and April the wind scale was more erratic. In the other months, the wind scale was generally category 1 or 2. Visibility varied greatly throughout the study period. The average value was about 12.5 km, while the maximum value was 29.5 km and the minimum value was 1.0 km. The average pressure in July was the lowest with an average of 98.7 kPa then from August to December it rose slowly, while from December to July it gradually decreased. Total sunshine intensity varied greatly throughout the study period. The average value was about 15.85 J/m^2^, while the maximum value was 28.59 J/m^2^ and the minimum value was 0.66 J/m^2^. The precipitation fluctuated greatly from February to May, with an average of 46.2%. Finally, the average daily emissions of industrial waste gas over the whole study period were 153 million cubic meters, while the maximum value was 270 million cubic meters and the minimum value was 100 million cubic meters. Average daily emissions of industrial waste gas in 2019 were 33 million cubic meters and 85 million cubic meters more than in 2018 and 2017, respectively, but the AQI annual average in 2019 was lower than both 2018 and 2017. Because Shandong Province implemented several air pollutant emission standards (“Emission standard of air pollutants for building materials industry”, Effective January 1, 2019) (“Emission standard of air pollutants for industrial furnace and kiln”, Effective June 1, 2019) in 2019, stricter pollutant emission concentration limits were implemented.

### AQI and variation correlation analysis

To verify that meteorological factors and industrial waste gas emissions affect air quality, we conducted a correlation analysis of AQI, meteorological factors, and industrial waste gas emissions in this paper, with the results presented in Table 2. Results indicate that industrial waste gas emissions were positively correlated with AQI, while visibility were negatively correlated with AQI. A rise in industrial waste gas emissions leads to an increase in AQI and the deterioration of air quality. As the amount of particulate matter in the air increases, it leads to the occurrence of haze and reduces visibility. There is a negative correlation between AQI and precipitation in the year and most seasons. This is because raindrops in the cloud can absorb and absorb pollutant particles, and at the same time, rainwater can wash and wash pollutants, resulting in lower pollutant concentrations, improved air quality, and lower AQI values. The correlation in winter is not obvious, which may be due to less precipitation in winter and uneven spatial and temporal distribution. There is a positively correlation between AQI and air temperature in the year and most seasons. From the seasonal scale, there is no obvious correlation between AQI and air temperature in spring. AQI in summer is significantly positively correlated with air temperature, which may have a certain relationship with the activity of cold and warm air masses, because when warm air masses pass through, the temperature will increase and a large amount of pollutants will accumulate. When the cold air passes through, it will reduce the temperature and often accompanied by wind, which is conducive to the diffusion of pollutants. The activities of cold and warm masses often occur frequently in summer. In autumn, atmospheric turbulence activities will intensify with the increase of air temperature, which will dilute and diffuse pollutants in the vertical direction of the lower layer, and further lead to the decrease of AQI. While rises in temperature can cause the temperature inversion phenomenon in winter, and exacerbating the air pollution problem. Ye’s analysis of Fairbanks confirmed that air temperature and AQI were positively correlated, while visibility was negatively correlated with AQI^28^. Guo studied the correlation between meteorological factors and AQI and also verified that there was a positive correlation between temperature and AQI^29^. These are consistent with the correlation analysis results obtained in this paper.

**Table 2:** Correlation between seasonal and annual AQI and meteorological elements from 2017 to 2019.

Season	Air temperature	Wind scale	Visibility	Air pressure	Total sunshine intensity	Relative humidity	Precipitation	Industrial waste gas emissions
Spring	0.034	− 0.084	− 0.513	− 0.042	0.010	− 0.018	− 0.252	0.224
Summer	0.227	0.047	− 0.189	− 0.025	0.347	− 0.363	− 0.426	0.587
Autumn	− 0.292	− 0.063	− 0.610	0.210	− 0.248	0.068	− 0.34	0.252
Winter	0.456	− 0.208	− 0.646	− 0.353	− 0.164	0.498	0.129	0.315
Year	0.354	− 0.165	− 0.526	0.293	− 0.215	− 0.012	− 0.326	0.374

Results indicate that the correlation of AQI with other meteorological elements (relative humidity, wind level, Air pressure, Total sunshine intensity and precipitation) is not the same on different time scales, because these meteorological elements vary greatly on different time scales. Taking relative humidity as an example, different scholars have studied the relationship between urban air pollution characteristics and meteorological conditions, and found that some cities have a positive correlation between pollutant concentrations and relative humidity^30–33^, and some cities have a negative correlation with relative humidity^28,34–38^. In Zhangdian District, there are different correlations between AQI and relative humidity in different seasons. There is an obvious positive correlation in winter, a negative correlation in summer, and no correlation in spring and autumn. Under low humidity conditions, the growth of condensation nuclei in the atmosphere aggravates pollution, and under high humidity conditions, it will have a scavenging effect on pollutants due to deposition^39^. On the other hand, relative humidity is negatively correlated with AQI. The reason may be that when the relative humidity is low, it is often accompanied by strong winds, which is easy to cause sand and dust weather and make the air quality worse. It can be seen that relative humidity is not the dominant factor affecting the development of pollution, and comprehensive judgments need to be combined with pollution emissions, meteorological conditions, and chemical processes.

### Results of the random forest model

The data samples selected in this paper include meteorological factors (average temperature, wind scale, relative humidity, and visibility), industrial waste gas emissions, and AQI in Zhangdian District. In this study, we obtained a total of 1095 sets of data, among which 1064 sets of data were used as the training data for the AQI prediction model. The final 31 sets were used as test data to verify the model. The prediction process of the random forest model was implemented using the Python programming platform. In the Python program, we used the “RandomizedSearchCV” function to approximate the random forest algorithm parameters. Then, the “GridSearchCV” function was used to accurately search the parameters of the random forest. The optimal parameters that we obtained are presented in Table 3.

**Table 3:** Optimal parameters of the random forest model.

Parameter	Value
max_depth	60
max_features	5
min_samples_leaf	2
min_samples_split	5
n_estimators	1400

The random forest model was established after searching the optimal parameters of the random forest. The last 31 sets of original data were used as samples for prediction. The AQI prediction results are displayed in Table 4, which shows that the predicted AQI values are similar to the measured values, indicating that the predicted results are accurate.

**Table 4:** AQI prediction results of random forest model.

Date	Measured AQI values	Predicted AQI values
1th Dec.	87	92
2th Dec.	62	67
3th Dec.	79	85
4th Dec.	112	99
5th Dec.	81	91
6th Dec.	79	90
…	…	…
26th Dec.	92	99
27th Dec.	71	86
28th Dec.	80	102
29th Dec.	69	137
30th Dec.	105	98
31th Dec.	54	61

**Figure 6:**
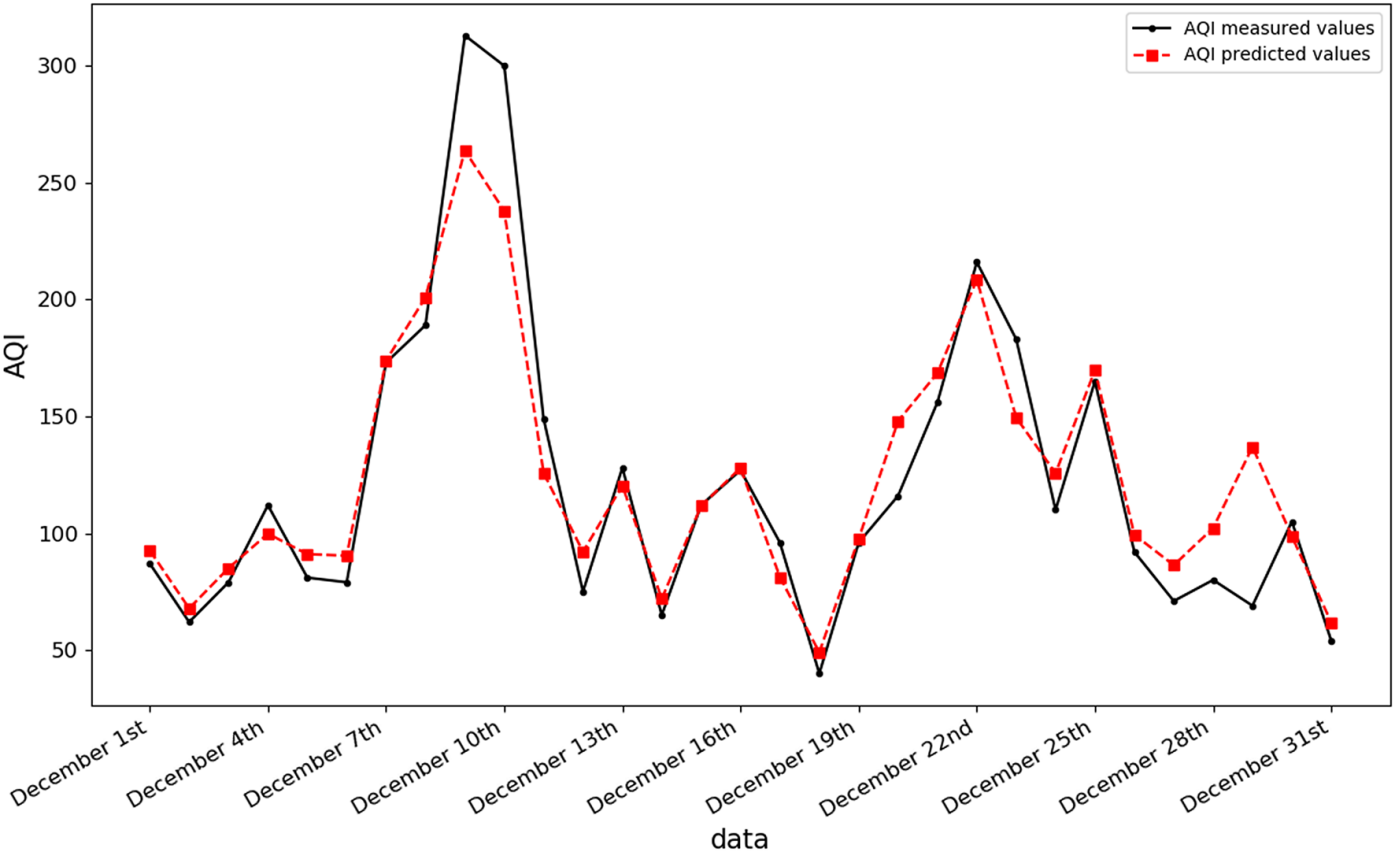
Comparison of modelled and measured AQI values.

**Figure 7:**
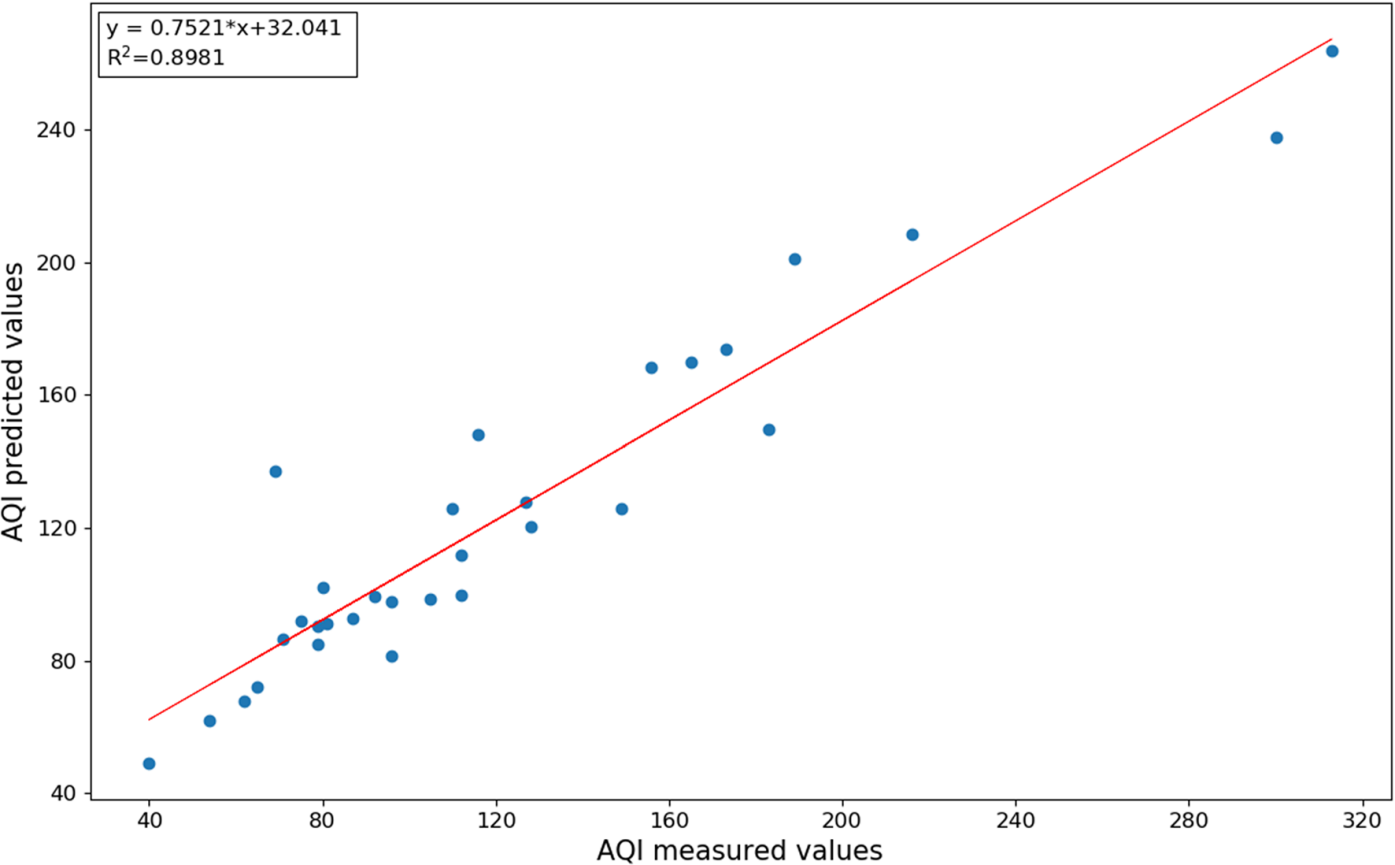
Linear fitting of predicted and measured AQI values.

The AQI predicted by the random forest model was compared with the measured AQI. It can be seen from Fig. 6 that the trend of the predicted and measured AQI is fundamentally the same. Figure 7 illustrates that the R2 value is 0.90, and the scatter points are precisely distributed at both ends of the line, indicating that the linear fitting is accurate. We can conclude that in this region it is effective to use meteorological factors and daily emissions of industrial waste gases to predict the AQI.

### Variable importance evaluation

**Figure 8:**
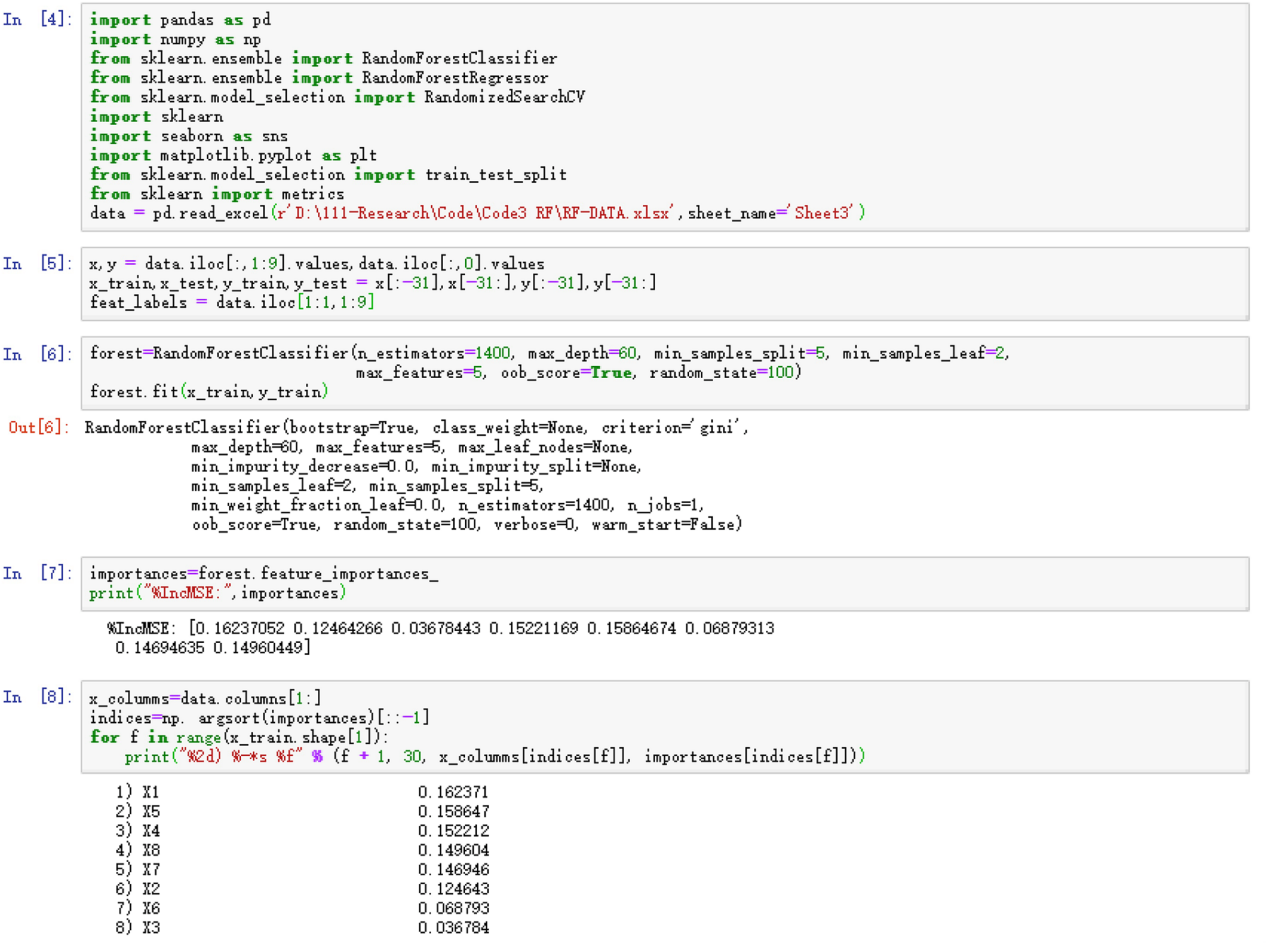
Python code for importance evaluation calculations.

In this study, we used Python to calculate the mean square residual (%IncMSE) in the random forest algorithm and determine the importance of each input variable. The Python code and parameters are presented in Fig. 8. A larger mean square residual reduction value indicates that the input variable has a larger influence on the output variable. As shown in Table 5, industrial waste gas (X1) was the greatest variable affecting AQI, followed by visibility (X5), relative humidity (X4), total sunshine intensity (X8), air pressure (X7), air temperature (X2) and precipitation (X6). The mean square residual value of the wind scale (X1) is the smallest, indicating that the influence of the wind on AQI is negligible compared with the other variables.

**Table 5:** Tanking of importance of variable.

Input variable	%IncMSE
X1	0.162371
X5	0.158647
X4	0.152212
X8	0.149604
X7	0.146946
X2	0.124643
X6	0.068793
X3	0.036784

### Model prediction accuracy evaluation

By comparing the random forest algorithm with other machine learning algorithms, we can verify the applicability of the random forest algorithm for air quality prediction in Zhangdian District. In this paper, four kinds of machine learning algorithms were used to predict AQI, and their results were compared to ascertain the most appropriate machine learning algorithm. The RMSE, MAE, and R^2^ measures were used to evaluate the prediction accuracy of the four machine learning algorithms^28^. For these algorithms, lower RMSE and MAE values indicate higher prediction accuracy, while the closer the R^2^ value is to 1, the more accurate the prediction is. The results presented in Table 6 confirm that the prediction accuracy of the random forest model is better than the other three machine learning models, indicating that the random forest model is the most suitable algorithm for the AQI prediction model of Zhangdian District.

**Table 6:** Model prediction accuracy evaluation.

Model	RMSE	MAE	R^2^
Random forest	22.91	15.80	0.90
BP neural network	26.72	17.53	0.81
Decision tree	29.85	18.11	0.76
LSSVM	26.29	17.37	0.80

### Control of industrial exhaust emissions based on target AQI

It can be seen from Table 7 that the measured AQI value of this region on 9th December was 263 (heavy pollution), and the industrial waste gas emission on that day was 191.9 million m^3^. The modelled results show that if the daily industrial waste gas emissions were controlled at 72.8 million m^3^, the air quality of the day could reach an acceptable level (AQI = 100). Conversely, on 18th December, the measured AQI value of the region was 49. The local meteorological conditions were favourable on this day, so the production time of high-polluting manufacturing processes load could be appropriately increased, and the daily industrial waste gas emissions could be increased by 378.9 million m^3^.

**Table 7:** Target industrial emissions at AQI of 100.

Air pollution Date	Predicted AQI value	Daily emissions of industrial waste gas (10^8^ m^3^)	Daily emissions of industrial waste gas to reach target AQI of 100 (10^8^ m^3^)	Difference (10^8^ m^3^)
1th Dec.	92	2.027	2.188	− 0.161
2th Dec.	67	1.972	2.907	− 0.935
3th Dec.	85	1.960	2.304	− 0.344
4th Dec.	99	1.946	1.950	− 0.004
5th Dec.	91	1.868	2.052	− 0.184
6th Dec.	90	2.029	2.245	− 0.216
7th Dec.	174	1.855	1.067	0.788
8th Dec.	201	1.966	0.978	0.988
9th Dec.	263	1.919	0.728	1.191
10th Dec.	237	1.897	0.797	1.1
11th Dec.	125	2.029	1.613	0.416
12th Dec.	92	2.028	2.203	− 0.175
13th Dec.	120	1.997	1.658	0.339
14th Dec.	72	1.891	2.628	− 0.737
15th Dec.	112	1.912	1.712	0.2
16th Dec.	128	2.017	1.576	0.441
17th Dec.	81	1.869	2.301	− 0.432
18th Dec.	49	1.857	3.789	− 1.932
19th Dec.	98	2.011	2.060	− 0.049
20th Dec.	148	1.987	1.343	0.644
21st Dec.	169	1.980	1.175	0.805
22nd Dec.	209	1.885	0.904	0.981
23rd Dec.	149	2.023	1.353	0.67
24th Dec.	126	1.960	1.559	0.401
25th Dec.	169	1.955	1.152	0.803
26th Dec.	99	1.884	1.897	− 0.013
27th Dec.	86	1.967	2.279	− 0.312
28th Dec.	102	2.004	1.960	0.044
29th Dec.	137	1.880	1.372	0.508
30th Dec.	98	1.988	2.015	− 0.027
31th Dec.	61	1.922	3.110	− 1.188

It can also be seen from Table 6 that the air quality in this region was poor in December 2019. There were 4 days of heavy air pollution, 3 days of moderate air pollution and 9 days of mild air pollution. According to the rationality of meteorological conditions, the air quality in this area could be maintained in good condition (AQI < 100) by increasing or reducing the industrial exhaust emission. It can also be seen from Table 6 that the total allowable exhaust emission in this area would be decreased by 361 million m^3^ compared with the actual emission in December 2019. The production capacity of enterprises would be decreased, but it would be better than the direct shutdown. According to Zibo City’s Emergency Plan for Heavy Pollution Weather (implemented in 2021), if the air quality index is greater than 200, these 60 key enterprises will directly stop work and production.

There are a large number of ceramic factories in this region, and there are two main sources of exhaust gases in the production of ceramics. The first is dust from crushing, screening, granulation, and spray drying in the manufacture of preformed moulds, glaze materials, and colouring materials. The second is high-temperature flue gas containing S0_2_ and smoke produced in the operation of various kiln firing equipment. Due to the different operating times of each process in the different factories vary, the collective operational load and pollution load of the processes are not balanced. This leads to great fluctuations in the daily emissions of industrial waste gas. By reducing the scale of “firing” processes and appropriately increasing the level of “raw material preparation” or “moulding” in periods of adverse meteorological conditions, the daily emissions of industrial waste gas can be reduced to ensure that the local environmental air quality is maintained at an acceptable level. On the other hand, increasing the operation of “firing” processes in favourable weather can balance the requirements of enterprises, allowing them to reach production targets. Given this, factories could reasonably adjust their production processes depending on the coming meteorological conditions, especially adverse meteorological conditions, to ensure that the regional environmental air quality is preserved in an optimal state.

### Feasibility analyze of enterprise process adjustment

Because the production process of the enterprise has the characteristics of multi-section cooperation, multi-machine parallel, and random “fluctuation” and nonlinear interaction between unit sections, the production process network presents great complexity and uncertainty. Production scheduling optimization research has always been a research hotspot. But the current research mainly focuses on the aspects of profit maximization^40^, time constraints^41^, capital constraints^42^, resource constraints^43^, energy constraints^44^, and production equipment constraints^45^. This research provides a new idea for the optimization of production scheduling in industrial enterprises.

The operation time of each production section within the enterprise is different, and the load is not balanced, so the sections that run every day are also different. The production process of some heavy air pollution industries (surface coating, pharmaceuticals, packaging and printing, building materials production, etc.) has certain discrete and intermittent sections, such as magnetic pole smears in motor manufacturers, purification in pharmaceutical companies, and burning in architectural ceramics companies. into the waiting section. These polluting sections have the characteristics of discontinuous intermittent, and the operation time is flexible and adjustable.

Adjustment of polluting processes or sections in the enterprise: (1) Verify the contribution index or scale model of each polluting process or section of the enterprise to the overall emission of the enterprise; (2) Insert the model index into the ERP (Enterprise Resource Planning) self-made parts material scheduling module to convert the process capability; (3) the process capability is adjusted by bringing the environmental prediction index in one cycle into the process capability calculation.

## Conclusions

In this study, a random forest model is used to construct an air quality prediction model in Zhangdian District based on the real-time dynamic emission effect of industrial waste gas-meteorological conditions, and to quantify the impact of industrial waste gas on air quality in the region. Using this model, the daily emission limit of industrial pollution can be determined according to the weather forecast inversion, and the air pollution risk caused by unfavorable meteorological factors can be effectively avoided by adjusting the production capacity of the internal production process of the enterprise. This research actively responds to the “Fourteenth Five-Year Plan for National Economic and Social Development of Zhangdian District and the Outline of Vision 2035”: by promoting the implementation of typical production scenarios, empowering actions, focusing on digital industrial applications, using cloud computing, big data and other new-generation information technologies, and guidelines for building a new industrialized strong city in the country. It provides a new idea for Zhangdian District’s “14th Five-Year Plan” to achieve an average annual growth rate of regional GDP of more than 7% and the harmonious development of industry and environment.

## Data Availability

The datasets used and/or analysed during the current study are available from the corresponding author on reasonable request.
